# SFA-MDEN: Semantic-Feature-Aided Monocular Depth Estimation Network Using Dual Branches

**DOI:** 10.3390/s21165476

**Published:** 2021-08-13

**Authors:** Rui Wang, Jialing Zou, James Zhiqing Wen

**Affiliations:** 1 Digital Photoelectric Information Processing Technology Laboratory, School of Instrumentation and Optoelectronic Engineering, Beihang Uiversity, Beijing 100191, China; wangr@buaa.edu.cn; 2 Engineering Research Center for Intelligent Robotics, Ji Hua Laboratory, Foshan 528200, China; wenzq@jihualab.com

**Keywords:** monocular depth estimation, semantic segmentation, feature fusion, multi-task deep learning

## Abstract

Monocular depth estimation based on unsupervised learning has attracted great attention due to the rising demand for lightweight monocular vision sensors. Inspired by multi-task learning, semantic information has been used to improve the monocular depth estimation models. However, multi-task learning is still limited by multi-type annotations. As far as we know, there are scarcely any large public datasets that provide all the necessary information. Therefore, we propose a novel network architecture Semantic-Feature-Aided Monocular Depth Estimation Network (SFA-MDEN) to extract multi-resolution depth features and semantic features, which are merged and fed into the decoder, with the goal of predicting depth with the support of semantics. Instead of using loss functions to relate the semantics and depth, the fusion of feature maps for semantics and depth is employed to predict the monocular depth. Therefore, two accessible datasets with similar topics for depth estimation and semantic segmentation can meet the requirements of SFA-MDEN for training sets. We explored the performance of the proposed SFA-MDEN with experiments on different datasets, including KITTI, Make3D, and our own dataset BHDE-v1. The experimental results demonstrate that SFA-MDEN achieves competitive accuracy and generalization capacity compared to state-of-the-art methods.

## 1. Introduction

Depth estimation plays a fundamental role in numerous application scenarios, such as image reconstruction [1], object detection [2], semantic segmentation [3], pose estimation [4], and medical image processing [5]. There are various sensors available to obtain depth information based on either active or passive measurement methods. Time-of-Flight (TOF) [6] is an active sensor that computes depth by the amount of time the infrared ray takes to travel back and forth between the emitter and the camera. Structured light cameras can predict depth accurately, even in weak light conditions, but require complex calibration and expensive costs. Stereo vision systems [7], using the passive measurement method, achieve high-quality depth estimation from rich-textured images via the configuration of binocular or even multi-view cameras. However, the system structure parameters, such as focal length and baseline, can greatly affect the calibration precision of cameras, which limits the flexibility of the stereo vision system. Monocular depth estimation refers to the scene depth recovery from a single two-dimensional image captured by the camera [8], which benefits the development of lightweight sensors. Nevertheless, given a two-dimensional image, multiple three-dimensional scenes will be reconstructed, which indicates that monocular depth estimation is an inherently ill-posed problem. For the past few years, an emerging computational imaging technology, where deep learning technology constrains estimation by extracting additional features, has become a major approach for monocular depth estimation.

In this paper, a novel network architecture with two branches is proposed to couple the semantic segmentation feature into a monocular depth estimation network aimed at improving the robustness and precision of depth estimation models. Generally, for unsupervised models, a left-view image is fed into the network to generate the disparity map, which is also known as the inverse depth and could warp the left-view into a predicted right-view image. Then, the self-supervised loss signal is computed through the difference between the predicted and the ground truth right-view images. The estimated depth map can be established by depth = baseline × focal_length/in-verse_depth. In addition, unsupervised methods learn to simulate a binocular system with the intrinsic parameters of the training dataset during the process. Thus, unsupervised networks perform unsatisfactorily on other datasets.

Semantics and depth are different comprehension patterns of an image scene, but have close relations. Inspired by the work of Stathopoulou et al. [4] who constructed geometric constraints based on semantics to optimize reconstruction from multi-view images, we believe multi-task learning via effectively merging semantic segmentation and depth estimation can improve the robustness and generalization capacity of networks. Traditional multi-task learning for predicting depth and semantic segments simultaneously employs a multi-term loss, which requires not only semantic annotations, but depth self-labels referring to stereo images or image sequences from the training datasets. Unfortunately, there are small datasets available but no large public datasets satisfying these label requirements currently. In view of this, we decided to predict depth robustly and accurately, even without the corresponding result of semantic segmentation. By combining the semantic information in feature maps with the depth estimation network, we no longer require joint depth and semantic annotations from the training set. In brief, different datasets for semantics and depth, respectively, are adopted to train our network.

Obviously, how to effectively deploy semantic information for the improvement of monocular depth estimation performance is an open issue. To address the problem, we proposed a novel network architecture to break the severe restriction of dataset annotations; the main contributions of the current study are as follows:

1. We propose a Semantic-Feature-Aided Monocular Depth Estimation Network (SFA-MDEN), which fuses multi-resolution semantic feature maps into networks to achieve better precision and robustness for the monocular depth estimation task.
2. A training strategy termed as Two-Stages for Two Branches (TSTB) is designed to train the SFA-MDEN. Instead of using the paired semantic label and stereo images for the input, a dataset for semantic segmentation and another dataset for monocular depth estimation with similar themes will satisfy the requirement. Therefore, larger datasets are guaranteed for better use of the semantic information to train our network.
3. Our SFA-MDEN achieves competitive estimation not only on public datasets KITTI [9] and Make3D [10], but also on our self-built dataset BHDE-v1 (Beihang University Depth Estimation dataset—version 1), which is a sparse depth dataset created to validate the generalization of our network.

The remainder of the paper is organized as follows: Section 2 reviews existing monocular depth estimation models based on deep learning, especially the methods involving semantics; the structure and the TSTB training strategy of SFA-MDEN are presented in Section 3; experimental results and analysis are presented in Section 4; and, finally, Section 5 presents the concluding remarks.

## 2. Related Works

The method proposed by this paper couples semantic segmentation with monocular depth estimation networks based on deep learning. Therefore, we introduce the state of monocular depth estimation research and the improvement with semantic features.

### 2.1. Monocular Depth Estimation

Monocular depth estimation refers to predicting the depth map with only a single view at the test. In 2008, Saxena et al. [11] assumed depth value as a function from the image features and the related parameters of the model could be learned by a training set based on MRF (Markov random field). In 2016, the convolutional neural network (CNN) was applied to learn the parameters as well [12]. However, the assumed functions in these types of approach cannot handle all the situations, especially the thin structures. Moreover, the convincible global estimation is hard to reach for the depth values that are predicted locally. In the meantime, Karsch et al. [13] concentrated on generating the predictions which are more consistent in context with the input by copying the depth map from the training set. This approach, known as non-parametric scene sampling, demands not only that the entire training set is available at the test, but that the test scenes are similar to the training scenes. With the rapid development of GPU computing power and the explosive growth of attainable images, CNN has become a crucial solution for ill-posed issues recently. Therefore, monocular depth estimation based on deep learning, which is either a supervised method or semi-supervised and unsupervised method, has attracted increasing attention as well.

Supervised methods for monocular depth estimation can be categorized into two types: continuous regression problems and discretized ordinal regression problems. Eigen et al. [14] originally proposed to use the multi-scale feature information for continuous depth prediction based on CNN. The network consists of a Global Coarse-Scale Network, which estimates the depth structure of the whole scene, and a Local Fine-Scale Network for refining details. This approach does not rely on hand-crafted features and infers the depth value directly from the original input. Using the idea of multi-scale feature fusion, Eigen et al. [15] designed three scale networks, two of which performed local refinements. The study stated that the weights of the depth estimation network can be transferred to normal estimation and semantic segmentation; it revealed the connection among depth, normal and semantic. Later, end-to-end supervised learning for monocular depth estimation was developed to combine with the encoder–decoder structure. In the work of Laina et al. [16], the powerful feature extraction function of ResNet50 [17] is designed as an encoder and its final full connection layer is replaced by a decoder, which is composed of upsampling operation and convolution layers. Other researchers selected MobileNet v2 and DPN-92 as the encoder to reduce the number of network parameters and pass valid information [18,19]. In addition, Lee et al. [20] designed local planar guidance layers in the decoder, which guided the decoding of different spatial resolution feature maps from the encoder to generate the desired depth.

Depth variation in a scene is usually randomly distributed. The prediction of extreme depth in autonomous driving scenarios is usually quite difficult for both algorithms and human perception. Therefore, some researchers regard the monocular depth estimation as an ordinal regression task. Continuous depth is discretized into a large number of intervals, and pixels are classified into corresponding depth intervals by means of network learning [21]. Nevertheless, such algorithms have to face problems as follows. Firstly, there are tight connections between different depth interval categories for the consecutiveness of depth variation. Furthermore, depth discretization brings about quantization error and swing for estimated depth. Subsequently, Su et al. [22] proposed a soft-regression model which regresses by a scaling factor and uses an offset term to adjust the depth estimation and reduce the quantization error. To achieve fast ordinal classification, Kim et al. [23] proposed another lightweight structure network L-E Net.

To solve the requirements of the monocular depth estimation on the datasets, some scholars have begun to study semi-supervised methods. Kuznietsov [24] designed a multi-loss function that combines the supervised loss term, unsupervised geometric consistency loss, and a regular term. In addition to using sparse depth information, synthetic data are involved in the following research papers. Amir et al. [25] proposed using a synthetic image library to train a monocular depth estimation network. To compensate for the domain gap, a style transfer network was designed. Zhao et al. [26] proposed a two-way style transfer network geometry-aware symmetric domain adaptation (GASDA) with multiple weighted losses, including bidirectional style transfer loss terms and depth estimation terms. The training process is cumbersome and puts severe requirements on the training datasets. Synthetic images are an alternative to real images, but they are still difficult to obtain. Directed against this problem, Ji et al. [27] proposed a new semi-supervised network framework, which includes a generator and two discriminators, to train the networks through a small number of high-quality RGB-D image pairs.

Because there is no need for deep labels, self-supervised learning has attracted the attention of scholars. According to the input images for training, self-supervised learning can be categorized into two classes: based on stereo image pairs and based on image sequences. Algorithms based on stereo image pairs were firstly proposed by Garg et al. [28] who adopted the encoder–decoder architecture to estimate the inverse depth map and generated the self-supervised loss by warped stereo views. Not only is a novel idea presented that does not rely on the ground truth depth during the training process, improvements on the loss functions and network structures are also developed in this research [28]. The left–right consistency loss is proposed by Godard et al. [29] to ensure structure similarity of bidirectional disparities. Following [29], Goldman et al. [30] constructed a Siamese network to predict bidirectional disparities. Inspired by the idea of the image scale pyramid, Poggi et al. [31] designed PyD-Net for real-time use. In recent years, Ye et al. [32] introduced a dual-attention module to enhance feature representations. Attention mechanism has also been researched to improve the edges of depth maps [33] and general contextual information [34].

The unsupervised methods based on stereo image pairs simulate the stereo vision system, utilizing neural network learning, and create a virtual binocular system [35] through monocular images, while the other type of unsupervised method based on image sequence simulate the camera movement through multi-frame images. Zhou et al. [36] proposed an end-to-end approach, where the supervision signal comes from the left and right adjacent frames, which are warped to the target frame based on the perspective projection model, and the training cost is computed by comparing the warped frame with the real target frame. Afterward, some improvements are achieved on this basis. To encourage the consistence of multi-frames, the three-dimensional geometry loss terms [37] and the traditional reconstruction loss terms [38] are put forward successively. In the last two years, there have also been some advances in estimating the depth of occluded and moving objects. Godard et al. [39] proposed Monodepth2, which estimated accurate depth for occluded object boundaries using the minimum reprojection loss and ensured camera motion assumptions through an auto-masking loss filtering out pixels of the dynamic objects, which move at the same velocity as the camera. Inspired by [39], as for occlusions, Ramamonjisoa et al. [40] designed a displacement field to resample pixels of occlusion boundaries. Concerning object motions, structure constraints about the object size [41] are introduced to model objects in 3D, and a self-discovered mask [42] is designed to infer moving objects automatically. As for real-time performance, Liu et al. [43] designed MiniNet with a periodic module to reduce network parameter. Wang et al. [44] designed a depth normalization layer to handle the generation of smaller depth values, which are caused by the commonly used smoothness items [29]. Additionally, Yin et al. [45] proposed the GeoNet, which joins learning of monocular depth, optical flow, and ego-motion with an adaptive geometric consistency loss.

### 2.2. Monocular Depth Estimation with Semantics

As mentioned earlier, semantics and depth are the scene understanding of different patterns, so some scholars are also committed to improving monocular depth estimation with semantic guidance. Research in this area is mainly developed in the manner of multi-task learning. Cpolla et al. [46] proposed training networks for semantic segmentation, depth estimation, and instance segmentation jointly. Ramirez et al. [47] proposed adding a supervised semantic segmentation decoder so that the encoder can learn features that are conducive to semantics. Mousavian et al. [48] used a fully connected CRF to capture the contextual relationship between semantic cues and depth cues to improve the accuracy of the final result. These methods put strict requirements on the training sets, requiring the input image to have a multitude of different types. For the joint tasks of semantic segmentation and depth estimation, both depth labels and pixel-by-pixel semantic annotations are necessary. However, there are very few large public databases that meet this requirement. To solve this very problem, Nekrasov et al. [49] proposed using the expert system trained on other semantic corpus to perform semantic segmentation on the datasets with depth labels in order to balance the number of annotations. Additionally, Yue et al. [50] proposed the semantic monocular depth estimation network SE-Net, which includes one part for estimating the preliminary depth map and another part for estimating a semantic weight map, which aims to adjust the preliminary prediction and construct a final depth map. The two parts are trained on the different datasets, and the result of semantic segmentation is used as a post-process step for depth map optimization. The above shows that methods with semantic supports are trapped in the dilemma of severe requirements on datasets that semantic annotations and corresponding depth labels are necessary simultaneously.

In this paper, based on the encoder–decoder structure, we propose a dual-branch framework which fuses semantic features into the monocular depth estimation network without requiring the ground truth depth. Compared to existing methods, we train the network through a TSTB strategy based on two different datasets with a similar theme to overcome the limitation of datasets, which effectively avoids the data acquisition dilemma.

## 3. Methodology

In this section, we introduce the framework and training strategy of SFA-MDEN, which predicts the depth from a single image with semantic guidance.

### 3.1. Framework and Training Strategy

As mentioned previously, monocular depth estimation with semantic optimization demands semantic corpus and stereo pair sets in a large public dataset, which is unapproachable. Fortunately, there are available training datasets for depth prediction and semantic segmentation, respectively. SFA-MDEN is designed to implicitly couple semantic features into depth estimation without requiring two correspondent annotations (stereo image pairs are also thought of as depth annotations in a sense).

**Figure 1:**
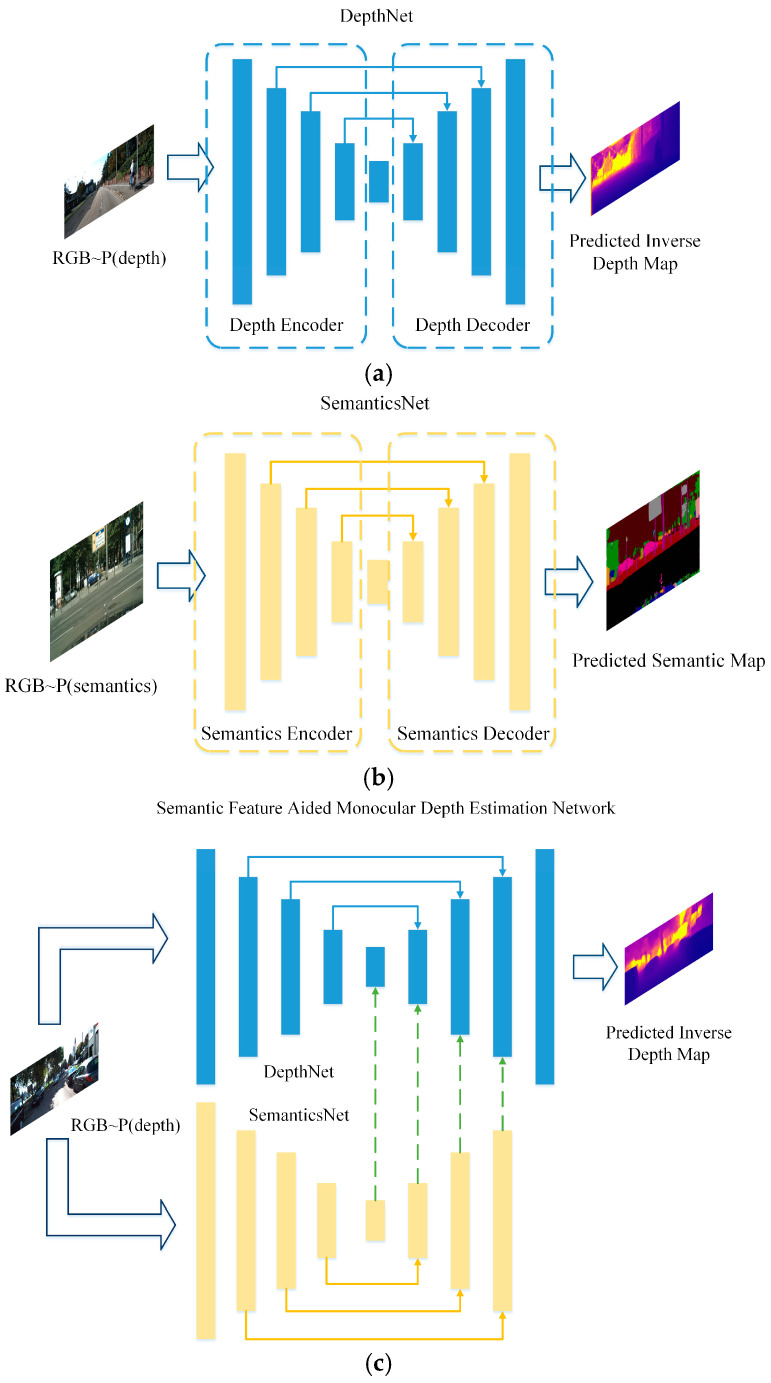
Overview of SFA-MDEN. The symbol RGB~P(depth) means that the input RGB image is required to have depth annotations for training. Similarly, RGB~P(semantics) means the requirement of semantics labels. (**a**) DepthNet: an unsupervised network to predict depth map as the main branch of SFA-MDEN, (**b**) SemanticsNet: a network for semantic segmentation to generate multi-resolution semantic feature maps for SFA-MDEN, (**c**) The overall framework of SFA-MDEN

The backbone of SFA-MDEN consists of an unsupervised monocular depth estimation network DepthNet and a supervised semantic segmentation network SemanticsNet, as shown in Figure 1a,b. Both networks adopt the encoder–decoder architecture with long-skip connections as the backbone. Depth differs from semantics, but there exists an implicit and tight interplay between them. Adjacent pixels are segmented to different semantic areas, which often means that occlusion, lighting changes, etc., have occurred, and usually result in a significant fluctuation in depth. Adjacent pixels segmented to the same semantic areas represent a single object, of which the depth usually varies continuously. Feature maps from the SemanticsNet reflect the depth fluctuation from another view. Therefore, we merge the corresponding scale feature maps in the dual branches and decode them to construct the final depth map, as shown in Figure 1c.

Aiming to break the limitation that the input image is required to have a multitude of different types of annotations, a Two-Stages for Two Branches (TBTS) training strategy is designed, which trains the branches independently and incorporates the two branches into a single network. In our SFA-MDEN, the DepthNet and SemanticsNet are the two branches. In Stage 1, DepthNet and SemanticsNet are trained separately using different datasets. For DepthNet, we use the KITTI datasets for training to obtain the initial weight of DepthNet in Stage 2. For SemanticsNet, the CityScapes [51] corpus is used to enable the Semantics Encoder to extract multi-scale semantic features. In Stage 2, semantic feature maps and depth feature maps in the corresponding scale are cascaded in the channel dimension. The details of the networks are introduced in Section 3.2. The parameters of Semantics Encoder are frozen and can extract semantic features from different scales. Semantics Decoder and DepthNet employ the parameters obtained in Stage 1 as the initial weights, and are trained on the KITTI datasets to achieve monocular depth estimation combined with semantic features.

Based on the TSTB training strategy, our SFA-MDEN has achieved monocular depth estimation based on the stereo sets of KITTI and semantic corpus of Cityscapes.

### 3.2. Network Architectures

**Figure 2:**
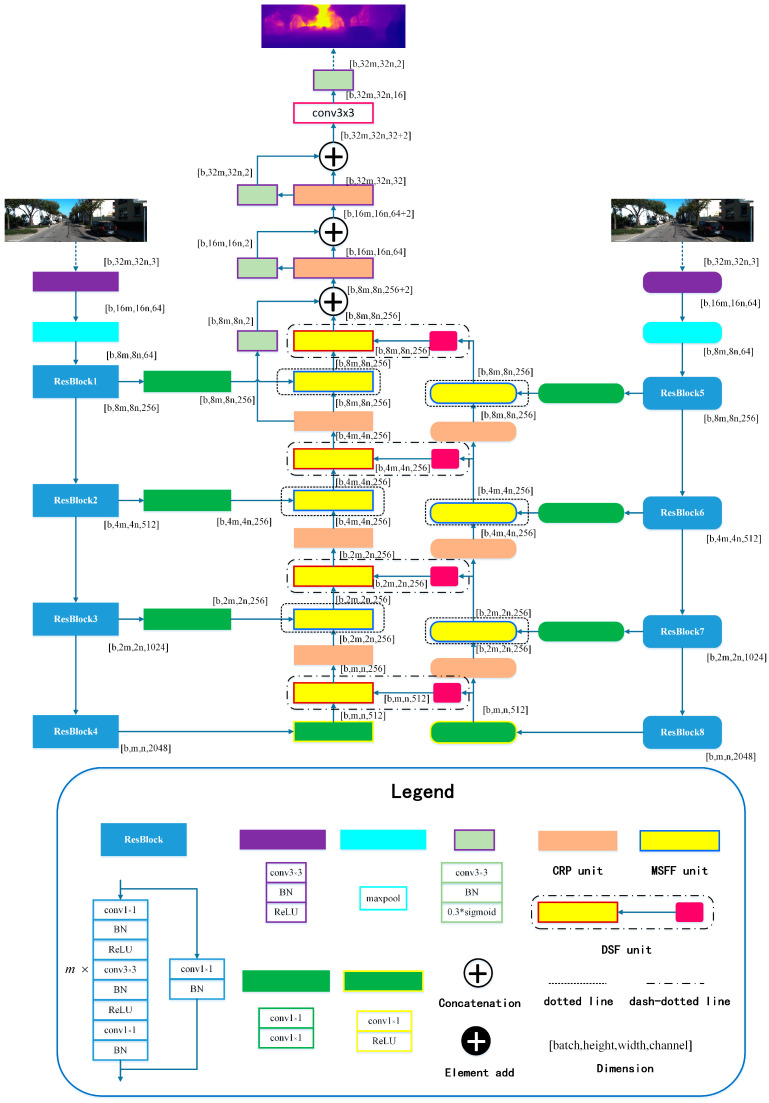
Our proposed architecture, named SFA-MDEN, exploits depth features and semantic features at different levels and fuses them to obtain a high-resolution monocular depth estimation. The square-corner rectangles denote the component modules of DepthNet, while the rounded rectangles denote the components of SemanticsNet. Structures of some components are illustrated in the form of a legend and some are shown in Figure 3. See text and Figure 3 for more details.

**Figure 3:**
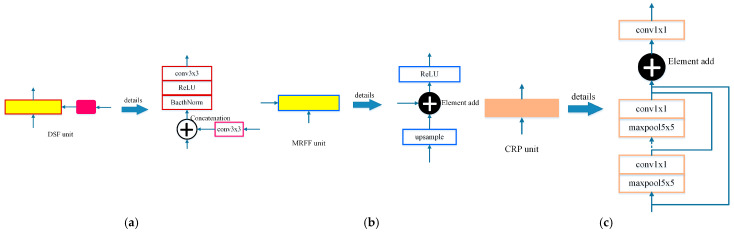
(**a**) DSF unit, (**b**) MRFF unit, and (**c**) CRP unit. In each illustration, the left block is the representation of the module in Figure 2 and its detailed structures are on the right. The yellow blocks denote the feature fusion modules. The red edge denotes the fusion of different types of features with the magenta block of the adaptive convolution and the blue edge denotes the fusion of features with different resolutions. The CRP unit is represented as the orange block.

Theoretically, the architecture of SFA-MDEN can be applied to any encoder–decoder structure. In this work, Light-Weight RefineNet [52] is adopted as the backbone. Additionally, ResNet50 without the fully connected layer is applied as the encoders. The main architecture of SFA-MDEN is shown in Figure 2. The input image is fed into Depth Encoder and Semantics Encoder separately. The two encoders, which are composed of ResBlocks from ResNet50, extract feature maps with four different resolutions, including 1/4, 1/8, 1/16, and 1/32, with the baseline of the input image. The detailed structures of partial modules are presented in the form of a legend, and the important modules are illustrated in Figure 3.

**Depth–Semantics-Fusion (DSF) unit.** The DSF unit, as shown by the dash-dotted line in Figure 2, achieves the fusion between different types of feature maps with the same resolution, with detailed structure in Figure 3a. The semantic feature maps generated by the Semantics Encoder are fine-tuned through an adaptive convolution operation for the monocular depth estimation task. The adaptive semantic features differ from depth features in geometric expression. Therefore, concatenation in channel dimension is adopted for the feature map fusion. BatchNorm layer is introduced to adjust the distribution of concatenated feature maps to avoid gradient vanishing and exploding. After the activation operation of ReLU, the feature group passes a convolution and the two types of features are fused to hunt for the new features.

**Multi-Resolution Feature Fusion (MRFF) unit.** The MRFF unit is employed to fuse the same type of features with different resolutions, as shown by the dotted line in Figure 2. The detailed structure is illustrated in Figure 3b. The lower resolution feature maps are bilinear, interpolated to be consistent with the higher resolution. The same type of feature maps in the corresponding channel are similar in geometric structure, and feature combination is carried out by adding items in an element-wise manner. After the feature maps are fused and activated, the feature map content of each channel increases.

**Chained Residual Pooling (CRP) unit.** The CRP unit [46] is established as a chain of pooling sub-blocks, each of which contains a max-pooling layer and a convolution operation, as denoted in Figure 3c. For each pooling sub-block, the output of previous blocks is taken as the input. Therefore, the CRP unit could pool features with multiple window sizes and fuse them together via the manner of element adding. Thus, it can capture information from a large region of the fused features and promote decoding.

### 3.3. Loss Function

Our SFA-MDEN is trained through the bidirectional loss items on four scales, including full, half, quarter, and eighth resolution. The bidirectional disparities contain the disparity from right to left view, denoted with superscript *l*, and the disparity from left to right view, denoted with the superscript *r*. The bidirectional loss on a single scale s consists of three weighted contributions as defined in Equation (1). The loss items are computed based on the bidirectional disparities. k denotes the corresponding weight with $k_{re}=1,k_{ds}=0.1,k_{lr}=1$. Additionally, the loss function of DepthNet is the same.

(1) $$L_{s}=k_{re}(L_{re}^{l}+L_{re}^{r})+k_{ds}(L_{ds}^{l}+L_{ds}^{r})+k_{lr}\cdot (L_{lr}^{l}+L_{lr}^{r}),s=1,2,3,4$$

The first term, defined as Equation (2), denotes the image reconstruction error by computing the difference between the original left view $I^{l}$ and the warped image ${\tilde{I}}^{l}$. N in Equations (2)–(4) denotes the number of pixels with valid depths.

(2) $$L_{re}^{l}=\frac{1}{N}\sum_{i,j}\frac{\tau }{2}[1-SSIM(I_{i,j}^{l},{\tilde{I}}_{i,j}^{l})]+(1-\tau )\left\|I_{i,j}^{l}-{\tilde{I}}_{i,j}^{l}\right\|$$

The disparity smoothness term, as (3), is deployed to encourage the gradient variation of the estimated disparity to approximate that of the input image. $\delta_{x}$ and $\delta_{y}$ represent the image gradients along the width and the height, and d denotes the estimated disparity in Equations (3) and (4). The gradient discontinuity loss is decayed by an exponential weight when corresponding discontinuity occurs on the input.

(3) $$L_{ds}^{l}=\frac{1}{N}\sum_{i,j}\left|\delta_{x}d_{i,j}^{l}\right|e^{-\left\|\delta_{x}I_{i,j}^{l}\right\|}+\left|\delta_{y}d_{i,j}^{l}\right|e^{-\left\|\delta_{y}I_{i,j}^{l}\right\|}$$

The last term encourages the consistency of the predicted bidirectional disparities, defined as Equation (4). A warped left-view disparity, constructed by projecting the right-view disparity, is encouraged to be coherent with the estimated left-view disparity.

(4) $$L_{lr}^{l}=\frac{1}{N}\left|d_{i,j}^{l}-d_{i,j+d_{i,j}^{l}}^{r}\right|$$

As for the training of SemanticsNet, we use the CrossEntropyLoss function, as defined in Equation (5). M denotes the number of categories, $y_{c}$ is the one-hot vector to indicate the ground truth, while $p_{c}$ is the probability that the sample falls into the category c.

(5) $$L_{semantics}=-\sum_{m=1}^{M}y_{c}\log(p_{c})$$

## 4. Experiments and Analysis

In this section, we compare our SFA-MDEN with existing supervised methods, self-supervised methods, and methods with semantic assistance. We consider two datasets for training, KITTI and Cityscapes. To evaluate our models, we test on the KITTI eigen split and analyze the performance of the model. In particular, to evaluate the estimation quality of relative location between pixels, two metrics, named Scale Invariant Error (SIE) and Absolute Relative Error of Relative Depth (ARERD), are computed. Meanwhile, we test the model directly on the Make3D datasets without any fine-tuning to validate the generalization performance of SFA-MDEN. Finally, a self-made dataset BHDE-v1 is applied to further evaluate the generalization ability and robustness of SFA-MDEN.

### 4.1. Implementation Details and Metrics

According to the TSTB training strategy, the training process is divided into two stages. In Stage 1, we train the DepthNet and SemanticsNet on different datasets, respectively. For DepthNet, we split the dataset as Eigen et al. [14] to compare with existing works. There are 61 scenes in total, divided into a test split of 20 scenes containing 697 image pairs, a training set of 22,600 image pairs, and the validation set of the remaining 888 image pairs from the remaining 32 scenes. We use the Adam optimizer with the hyper-parameter $\beta_{1}=0.9$, $\beta_{2}=0.999$, and $\varepsilon =10^{-8}$ with learning rate α=0.0001 and a batch size of 4. We flip the input images with a chance of 50% and add color augmentation for data augmentation. The corpus of Cityscapes is applied to train the supervised. SemanticsNet. The corpus consists of 5000 high-resolution images with 19 categories of fine semantic annotations from 50 cities. The training set includes 2975 images, with another 500 for validation, and the remaining 1525 for test. We select the mini-batch SGD with a momentum parameter of 0.9 and the initial learning rate of $10^{-4}$ as the optimizer. Random crop, random mirror, and random scaling are applied to augment the training set. Stage 2 is the training of the overall SFA-MDEN. Only the training set of KITTI is used. The hyper-parameters are almost the same as the DepthNet in Stage 1. The learning rate is set as $10^{-4}$ for the first 30 epochs, and is halved every 10 epochs thereafter. The proposed SFA-MDEN is trained on a computer configured as: 2.20 GHz × 8, Intel(R) Core (TM) i7-8750H CPU, and a single GeForce RTX 2080 Mobile GPU card with 8GB RAM.

Similar to previous works, we adopt four error metrics and three accuracy metrics to evaluate the models firstly, as follows:

- Absolute relative error: $AbsRel=\frac{1}{N}\sum_{i=1}^{N}\frac{\left|{\hat{D}}_{i}-D_{i}\right|}{D_{i}}$
- Square relative error: $SqRel=\frac{1}{N}\sum_{i=1}^{N}\frac{{\left|D_{i}-{\hat{D}}_{i}\right|}^{2}}{D_{i}}$
- Linear root-mean-squared error: $RMSE(linear)=\sqrt{\frac{1}{N}\sum_{i=1}^{N}{\left|{\hat{D}}_{i}-D_{i}\right|}^{2}}$
- Logarithm root-mean-squared error: $RMSE(\log)=\sqrt{\frac{1}{N}\sum_{i=1}^{N}{\left|\log{\hat{D}}_{i}-\log D_{i}\right|}^{2}}$
- Threshold accuracy (%correct): $\delta =\max(\frac{D_{i}}{{\hat{D}}_{i}},\frac{{\hat{D}}_{i}}{D_{i}})<T$ (T is the threshold and can be assigned as 1.25, $1.25^{2}$, $1.25^{3}$)

where ${\hat{D}}_{i}$ denotes the predicted depth, $D_{i}$ denotes the ground truth depth, the subscripts *i* and *j* are the pixel index, and N represents the total number of valid pixels.

The aforementioned four error metrics assess the accuracy of the predicted depth for merely a single pixel but, in some application scenarios, the accuracy of the relative depth between pixels is given more attention, for instance, the detection system for the landing site of an unmanned aerial vehicle (UAV). Therefore, we also employ SIE, as shown in (6), which was designed as a loss function by Eigen et al. [14], to evaluate the performance of our network. *SIE* represents the mean square error of the relative depth between each pair of pixels in log domain. n represents the number of point pairs.

(6) $$SIE=\frac{1}{n}\sum_{i,j}{\left((\log{\hat{D}}_{i}-\log{\hat{D}}_{j})-(\log D_{i}-\log D_{j})\right)}^{2}$$

Inspired by *SIE*, we design another metric *ARERD*, as shown in (7), which refers to the relative error of the point-pair distance directly.

(7) $$ARERD=\frac{1}{n}\sum_{i,j}\frac{\left|{\hat{D}}_{i}-{\hat{D}}_{j}\right|-\left|D_{i}-D_{j}\right|}{\left|D_{i}-D_{j}\right|}$$

### 4.2. Eigen Split of KITTI

We compare the performance of the proposed SFA-MDEN with existing monocular depth estimation algorithms. For accuracy evaluation, ground truth depth values are generated sparsely by reprojecting 3D points from Velodyne laser into the left input color camera. To weaken the threats caused by the rotation of the Velodyne, the motion of the vehicle, and the surrounding objects, along with occlusions, the cropping scheme proposed by [14] is adopted, which contains approximately 58% in height and 93% in width of the image.

Table 1 shows the comparison results with the supervised methods [12,14], the unsupervised methods [28,29,36,37,45,50], and the estimation methods related to semantics [50] at 80 m and 50 m caps. First, our algorithm has achieved a clear improvement compared to the most listed supervised and unsupervised methods in all metrics under both depth caps, except Yue et al. [50]. The reason is that Yue et al. [50] established a semantic weight map to optimize the estimated depth map, which takes advantage of semantic information as well. Even so, there is a narrow margin between our SFA-MDEN and Yue et al. [50] in metrics of Sq Rel, RMSE log, and $\delta <1.25^{3}$. Furthermore, the Abs Rel of SFA-MDEN is 0.023 smaller, the RMSE of SFA-MDEN is 0.135 m smaller, and the threshold accuracy δ<1.25 is 2.9% higher compared to Yue et al. [50]. Second, SFA-MDEN achieves considerable advances by merging the feature maps from SemanticsNet compared to the original DepthNet. All error metrics have been prominently reduced. Not only is Abs Rel declined by 16.67%, but Sq Rel is also decreased by 6.25%. Moreover, RMSE and RMSE log are decreased by 7.54% and 9.24%, respectively. The three metrics of threshold accuracy are increased by about 1%. The effectiveness of semantic features for monocular depth estimation is demonstrated and the validity of SFA-MDEN can be supported powerfully.

**Figure 4:**
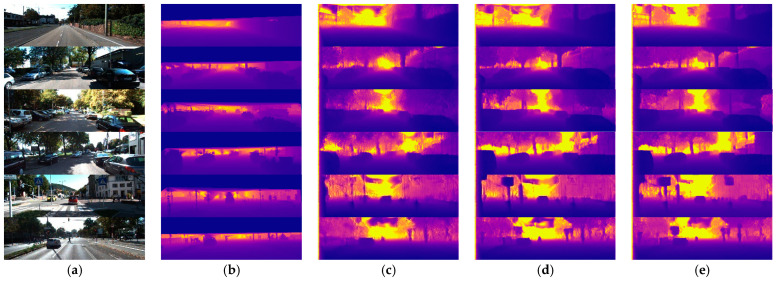
Qualitative samples on the Eigen split of KITTI. (**a**) The input RGB images, (**b**) ground truth depth maps, (**c**) outputs of Godard et al. [31], (**d**) outputs of DepthNet, (**e**) outputs of the proposed SFA-MDEN. Our method achieves the plausible estimation quality on the surfaces and boundaries.

In addition to the quantitative comparison, some qualitative samples are exhibited in Figure 4. The results of our SFA-MDEN are compared to Godard et al. [29] and DepthNet. According to the samples, the depth variation of the same object is more continuous, such as building surfaces with windows in outputs of SFA-MDEN. Furthermore, the depth variation of the object boundaries is more evident for our method. As shown in Figure 4, outlines of vehicles and road signs and are sharper and more plausible.

In addition to comparison with above methods, to illustrate the competitiveness of SFA-MDEN, we compare our method with other models with semantic guidance, as shown in Table 2. It should be noted that the training set and test set of these methods are not the same. First, Nekrasov et al. [49] use a teacher network to annotate semantic labels of KITTI to pretrain the target model. They have achieved outstanding results on the 46 test images of KITTI-6 on RMSE and RMSE log, even compared with methods in Table 1, which demonstrate the superiority of the semantic guide. Nevertheless, the expert systems can affect the accuracy of the model greatly and the whole process is tedious. Our SFA-MDEN does not depend on other models. Second, Ramirez et al. [47] pretrained their network on Cityscapes with about 3000 finely semantically annotated stereo images, fine-tuned the network on 160 stereo images with semantic labels of KITTI 2015, and tested on another 40 images. Observably, the model performs poorly due to the small dataset. We have compared Yue et al. [50] with our model quantitatively in Table 1. According to Table 2, generally speaking, SFA-MDEN performs the best estimation in the metric Abs Rel and $\delta <1.25^{2}$. Moreover, our model ranks second in Sq Rel and RMSE. Although SFA-MDEN comes last in RMSE log and $\delta <1.25^{3}$, there is a narrow margin between these methods. In conclusion, our method has achieved a competitive performance compared to the state-of-the-art methods with semantic auxiliary.

We can evaluate the models through diverse metrics and, in practice, we select the network according to the application. As previously mentioned, in certain application scenarios, such as the detection system for the landing site of UAVs, the precision of relative depth estimation accuracy between point pairs is of more concern. Therefore, the metrics of *SIE* and *ARERD* defined in Equations (6) and (7) are used to evaluate the models, as shown in Table 3. The model from Godard et al. [29] and DepthNet are selected to compare with SFA-MDEN due to their comparable performance on most metrics, as seen in Table 1. According to Table 3, our SFA-MDEN has achieved superior performance compared to the other two. Compared to DepthNet without the semantic information, SFA-MDEN achieves a decrease of 18.64% on the metric SIE and a decrease of 14.95% on the metric ARERD. We conclude that, after merging the semantic information into depth estimation, the accuracy of the positional relationship between pixels has been improved.

**Figure 5:**
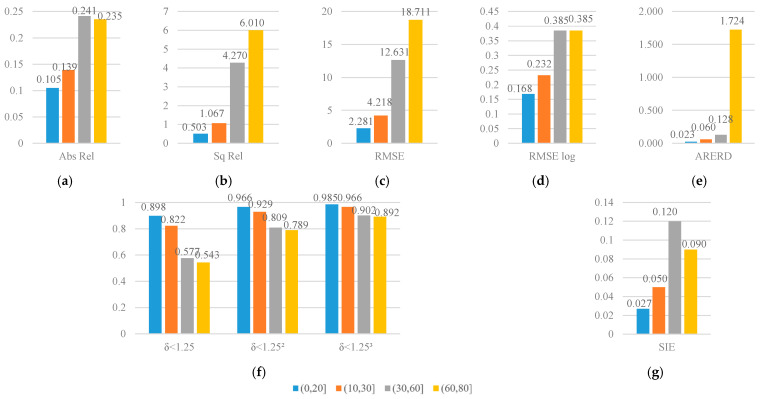
Quantitative comparison depth estimation precision in different depth ranges of the SFA-MDEN. (**a**) Abs Rel, (**b**) Sq Rel, (**c**) RMSE, (**d**) RMSE log, (**e**) ARERD, (**f**) accuracy metrics (δ<1.25, $\delta <1.25^{2}$, $\delta <1.25^{3}$), and (**g**) SIE.

The UAV landing system not only demands the accuracy of relative depth, but requires different accuracy with different working height according to the user guide of DJI UAV [53]. According to the user guide, there are four working depth intervals which are (0,20], (10,30], (30,60], (60,80]. Therefore, we evaluate the precision of pixels in the depth intervals and visualize the results as Figure 5. As shown in Figure 5, firstly, most trends in the metrics indicate that, as the depth increases, the prediction accuracy decreases. Secondly, Abs Rel shows a downward trend in the last two intervals. It can be interpreted that Abs Rel is defined as the absolute error divided by the ground truth depth value, and the absolute error of the estimation does not increase as much as the ground truth with the depth getting higher. Thirdly, SIE also shows a downward trend in the last two intervals. The reason can be explained as the large depth changes slower in the logarithmic domain, so the relative distance between the point pairs is smaller and the SIE is reduced.

### 4.3. Make3D

**Figure 6:**
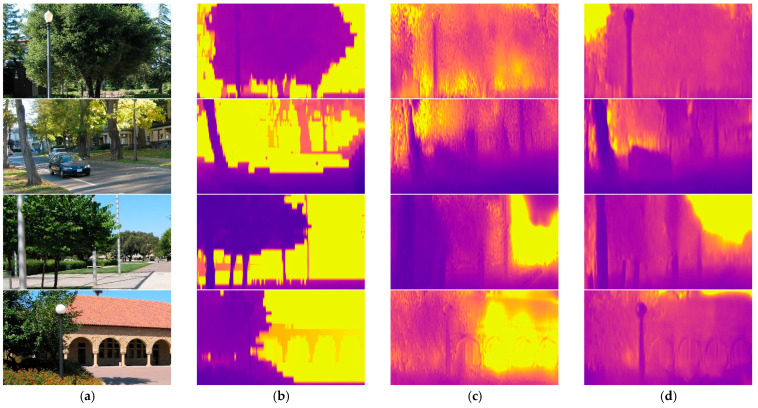
Qualitative samples on the Make3D datasets. (**a**) The input RGB images, (**b**) ground truth depth maps, (**c**) outputs of Godard et al. [29], and (**d**) outputs of the proposed SFA-MDEN.

Experiments on KITTI have indicated the estimation performance of SFA-MDEN. To evaluate the generalization of our SFA-MDEN, we compare it to other methods on the Make3D test set [10], which contains 134 images and the corresponding depth maps. Although our SFA-MDEN is trained on the KITTI and Cityscapes and is directly tested at a center crop of 2 × 1 ratio as [29] on the Make3D dataset, we still achieve comparable results, as shown in Table 4. First, we almost outperform all unsupervised methods, which illustrates the superiority of our SFA-MDEN. Second, compared to supervised methods which are trained with the ground truth depth of Make3D, the results of our SFA-MDEN without fine-tuning on the Make3D set are still comparable. Experimental results on the Make3D set show the strong generalization capacity of our SFA-MDEN even if it is applied to a different domain directly without fine-tuning. Qualitative results can be seen in Figure 6.

### 4.4. Self-Made Datasets: BHDE-v1

**Figure 7:**
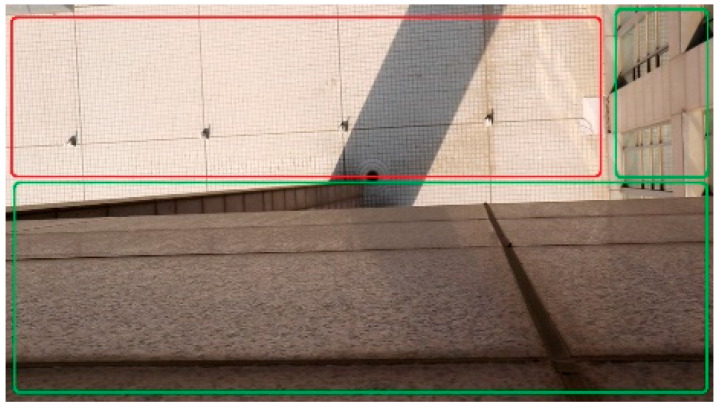
An image sample of the BHDE-v1 dataset. The area inside the red rectangle window is the sparsely depth-annotated plain land, while areas in the green window are not annotated.

In addition to the above public datasets, we captured a small dataset BHDE-v1 of, in total, 140 frames of images with the resolution of 1920×1080 in order to validate the generalization capacity of our SFA-MDEN. An image example is illustrated in Figure 7. The captured subject of the dataset is the plain land as the area in the red rectangle window dropping out the protrusions. We obtain the depth value from the camera to the plain land, which is 21.678 m, through the laser rangefinder GLM80. The measurement error of GLM80 is ±1.5 mm in the measurement range of 80 m, which is much less than that of the estimation from models. Thus, the measured depth can be viewed as the ground truth. Areas in the green rectangle windows are from the surrounding buildings with continuous depth, which we have not annotated.

**Figure 8:**
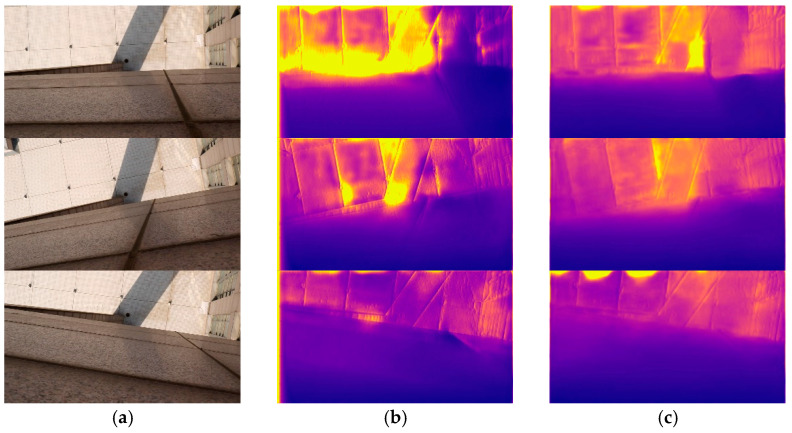
Qualitative samples on the BHDE-v1 dataset. (**a**) The input RGB images, (**b**) outputs of Godard et al. [29], and (**c**) outputs of the proposed SFA-MDEN.

SFA-MDEN and the model from Godard et al. [29], which performs well on the Eigen split of KITTI, are tested without fine-tuning on the BHDE-v1 dataset. Table 5 presents the quantitative results and Figure 8 illustrates the qualitative samples. As can be seen, the precision metrics of our SFA-MDEN are superior to Godard et al. [29], with a depth range of about 20 m. Moreover, SFA-MDEN indicates stronger robustness to the shadows and illumination, as shown in Figure 8. As for the floor gaps in the input image, the output of Godard et al. [29] shows significant depth changes, while our SFA-MDEN performs more robustly. In conclusion, our SFA-MDEN is verified to achieve better generalization and powerful robustness on different datasets.

## 5. Conclusions and Discussion

Monocular depth estimation based on deep learning achieves higher flexibility compared to binocular systems, which promotes the application of lightweight vision sensors. In this paper, inspired by multi-task training, a novel network architecture abbreviated as SFA-MDEN, which merges semantics-related features into the monocular depth estimation network, is proposed to predict the depth map for preferable precision, robustness, and generalization capacity. A Two-Stages for Two Branches training strategy is designed to train the SFA-MDEN on two large public datasets, KITTI for depth estimation and Cityscapes for semantic segmentation. In Stage 1, two branches are trained independently based on respective datasets, with the unsupervised DepthNet trained on the stereo sets of KITTI and the supervised SemanticsNet trained on the corpus of Cityscapes. In Stage 2, the SFA-MDEN is constructed by the feature fusion between semantics and depth and is trained on the stereo sets of KITTI, with the pretrained parameters of Stage 1 as the initial weights. At inference, the semantic features are extracted by the SemanticsNet branch and delivered to fuse with the depth feature extracted by the DepthNet branch. Experiment comparisons on the Eigen split of KITTI demonstrate the superiority of our proposed SFA-MDEN, even compared to existing methods with semantic auxiliary. Additionally, experiments on Make3D datasets have exhibited the generalization of SFA-MDEN, which is tested without fine-tuning, and experiments on our self-made datasets BHDE-v1 validate the robustness against the variation of shadow and illumination.

In future work, the relation between semantics and depth could be exploited further. Additionally, the loss function on account of traditional image reconstruction constraints is worthy of attention. Our future work will focus on improving the unsupervised monocular depth estimation with the semantic guidance in a lightweight network structure.

## Figures and Tables

**Table 1:** Monocular depth estimation evaluation using the split of Eigen et al. [14] on the KITTI dataset [9]. For datasets, K = KITTI, and CS = Cityscapes, CS + K means that the model is pretrained on the Cityscapes and finetuned on the KITTI. For methods with semantics, the dataset for semantic segmentation training is in brackets. We highlight the best results with the underline in each depth cap. The results of Garg et al. [28] are only capped at 50 m.

Method	Supervision	Semantics	Datasets	Cap (m)	Error Metrics				Accuracy Metrics		
					Abs Rel	Sq Rel	RMSE	RMSE log	δ < 1.25	$\delta \;<\;1.25^{2}$	$\delta \;<\;1.25^{3}$
Eigen et al. [14]	Depth	No	K	80	0.214	1.605	6.563	0.292	0.673	0.884	0.957
Eigen et al. Fine [14]	Depth	No	K	80	0.203	1.548	6.307	0.282	0.702	0.890	0.958
Liu et al. [12]	Depth	No	K	80	0.202	1.614	6.523	0.275	0.678	0.895	0.965
Yin et al. [45]	Sequence	No	K	80	0.155	1.296	5.857	0.233	0.793	0.931	0.973
Zhou et al. [36]	Sequence	No	K	80	0.208	1.768	6.856	0.283	0.678	0.885	0.957
Zhou et al. [36]	Sequence	No	CS + K	80	0.198	1.836	6.565	0.275	0.718	0.901	0.960
Mahjourian et al. [37]	Sequence	No	CS + K	80	0.159	1.231	5.912	0.243	0.784	0.923	0.970
Godard et al. [29]	Stereo	No	K	80	0.148	1.344	5.927	0.247	0.803	0.922	0.964
Yue et al. [50]	Stereo	Yes (CS)	K	80	0.143	1.034	5.505	0.219	0.809	0.937	0.977
DepthNet only	Stereo	No	K	80	0.144	1.168	5.808	0.249	0.828	0.930	0.963
SFA-MDEN	Stereo	Yes (CS)	K	80	0.120	1.095	5.370	0.226	0.838	0.939	0.972
Zhou et al. [33]	Sequence	No	CS + K	50	0.190	1.436	4.975	0.258	0.735	0.915	0.968
Mahjourian et al. [37]	Sequence	No	CS + K	50	0.151	0.949	4.383	0.227	0.802	0.945	0.974
Yin et al. [45]	Sequence	No	K	50	0.147	0.936	4.348	0.218	0.810	0.941	0.977
Garg et al. [28]	Stereo	No	K	50	0.169	1.080	5.104	0.273	0.740	0.904	0.962
Godard et al. [29]	Stereo	No	K	50	0.140	0.976	4.471	0.232	0.818	0.931	0.969
Yue et al. [50]	Stereo	Yes (CS)	K	50	0.137	0.792	4.158	0.205	0.826	0.947	0.981
DepthNet only	Stereo	No	K	50	0.140	1.010	4.724	0.239	0.812	0.925	0.966
SFA-MDEN	Stereo	Yes (CS)	K	50	0.116	0.956	4.361	0.217	0.848	0.948	0.974

**Table 2:** Comparison between monocular depth estimation methods with semantic auxiliary and SFA-MDEN. For datasets, K = KITTI, CS = Cityscapes, K-6 = KITTI-6, and K (200) denotes the 200 images Ramirez et al. [50] used in KITTI. We highlight the best results with an underline. Nekrasov et al. [43] only reported the evaluation metrics of RMSE and RMSE log.

Method	Supervision	Datasets	Cap	Error Metrics				Accuracy Metrics		
				Abs Rel	Sq Rel	RMSE	RMSE log	δ < 1.25	$\delta \;<\;1.25^{2}$	$\delta \;<\;1.25^{3}$
Nekrasov et al. [43]	Depth	K-6, K, CS	80 m	-	-	3.453	0.182	-	-	-
Ramirez et al. [40]	Stereo	CS, K (200)	80 m	0.143	2.161	6.526	0.222	0.850	0.939	0.972
Yue et al. [44]	Sequence	K, CS	80 m	0.143	1.034	5.505	0.219	0.809	0.937	0.977
SFA-MDEN	Stereo	K, CS	80 m	0.120	1.095	5.370	0.226	0.838	0.939	0.972

**Table 3:** Evaluation on the metrics of SIE and ARERD. We highlight the best results with an underline.

Method	SIE	ARERD
Godard et al.	0.051	0.0855
DepthNet only	0.059	0.0642
SFA-MDEN	0.048	0.0546

**Table 4:** Monocular depth estimation evaluation on the Make3D dataset. We highlight the best results with an underline, and bold numbers rank second in each metric.

Method	Supervision	Sq Rel	Abs Rel	RMSE	Log10
Karsch et al. [13]	Depth	4.894	0.417	8.172	**0.144**
Liu et al. [12]	Depth	6.625	0.462	9.972	0.161
Laina et al. [16]	Depth	1.840	0.204	5.683	0.084
Zhou et al. [36]	Sequence	5.321	**0.383**	10.470	0.478
Godard et al. [29]	Stereo	11.990	0.535	11.513	0.156
Wang et al. [44]	Stereo	4.720	0.387	8.090	0.204
SFA-MDEN	Stereo	**4.181**	0.402	**6.497**	0.158

**Table 5:** Monocular depth estimation evaluation on the BHDE-v1 of SFA-MDEN and the model in Godard et al. [29].

Method	Abs Rel	Sq Rel	RMSE	RMSE log	δ < 1.25	$\delta \;<\;1.25^{2}$	$\delta \;<\;1.25^{3}$
SFA-MDEN	0.142	0.562	3.266	0.167	0.775	0.959	0.980
Godard et al.	0.240	1.822	5.738	0.310	0.489	0.791	0.917

## Data Availability

The BHDE-v1 are available online at http://doip.buaa.edu.cn/info/1092/1113.htm (accessed on 5 August 2021).
